# Sustainable Digital Transformation: Its Impact on Perceived Value and Adoption Intention of Industry 4.0 in Moderating Effects of Uncertainty Avoidance

**DOI:** 10.12688/f1000research.152228.1

**Published:** 2024-07-22

**Authors:** Yuli Sartono, Endang Siti Astuti, Wilopo Wilopo, Teuku Noerman

**Affiliations:** 1 Faculty of Administrative Science, Brawijaya University, Malang, East Java, 65145, Indonesia

**Keywords:** digital trust, sustainable attitude, perceived value, intention to adopt industry 4.0, uncertainty avoidance

## Abstract

**Background:**

Industry 4.0 is a significant technical revolution that combines big data analytics, the Internet of Things (IoT), and cyber-physical systems to improve manufacturing productivity. This study investigates the impact of digital trust and sustainable attitude on perceived value and the intention to adopt Industry 4.0 technologies. It also examines the moderating role of uncertainty avoidance in these relationships.

**Methods:**

Data were collected from 189 employees of leading manufacturing companies in Indonesia that are recognized for their Industry 4.0 practices. The data were analyzed using Partial Least Squares (PLS) methodology with SmartPLS software to test the proposed hypotheses and explore the moderating effects.

**Results:**

The findings reveal that both digital trust and sustainable attitude significantly influence perceived value. However, these factors do not directly affect the intention to adopt Industry 4.0 technologies. Uncertainty avoidance moderates the relationship between digital trust and adoption intention. Specifically, in environments with high uncertainty avoidance, digital trust becomes a critical factor influencing the decision to adopt Industry 4.0 technologies.

**Conclusions:**

The study provides valuable insights for organizations aiming to implement Industry 4.0 initiatives. It highlights the importance of fostering digital trust and considering cultural dimensions, such as uncertainty avoidance, in their technology adoption strategies.

## 1. Introduction

Technology has played a pivotal role in human progress throughout history. Humans possess a natural inclination to innovate and create tools, which have been instrumental in shaping civilizations for millennia. Industry 4.0, hailed as a new industrial revolution, is driven by the evolving and diverse needs of society ( Tsaramirsis et al., 2022). It marks a significant shift in the way businesses and industries function, holding the potential to redefine the global economy’s trajectory ( Müller et al., 2018; Qian & Wang, 2022). Industry 4.0 entails the integration of digital technology, artificial intelligence (AI), big data, the Internet of Things (IoT), machine learning, and cloud computing to establish a smart, interconnected, and automated manufacturing landscape ( Ma et al., 2024; Javaid et al., 2022). Virmani et al. (2023) underscored the potential advantages of embracing Industry 4.0, such as enhancing operational efficiency and profitability through digital integration, elevating product quality, trimming production costs, and minimizing variations and defects, thereby bolstering overall operational efficacy.

According to a study conducted by Javaid et al. (2022), Industry 4.0 technology is recognized as playing a pivotal role in fostering innovation and driving future business growth. The implementation of Industry 4.0 has the potential to cultivate a sustainable environment ( Bonilla et al., 2018; Javaid et al., 2022), in part due to its contribution to energy efficiency and sustainability through the reduction of carbon emissions. Industry 4.0 offers principles, guidance, and technologies that facilitate the establishment of new factories and the enhancement of existing ones ( Ghobakhloo, 2018). This enables consumers to opt for diverse production models based on their requirements, with scalability that can be augmented through the utilization of advanced robotics, information technology, and communication systems. Furthermore, the study by Javaid et al. (2022) identifies twenty key applications of Industry 4.0 aimed at fostering a sustainable environment and providing deeper insights into production environments, supply chains, delivery channels, and market outcomes.

Industry 4.0 has introduced a profound transformation in the world of business and industry. However, the transition towards more connected and automated production models does not come without challenges. Organizations are faced with new complexities in managing more integrated systems, handling big data, and navigating rapidly changing environments ( Selten & Klievink, 2024; Wang et al., 2018). These challenges often give rise to uncertainty, which can influence strategic decisions regarding the adoption of Industry 4.0.

Additionally, the barriers to the implementation of Industry 4.0 include the scarcity of individuals with digital skills ( Koerber et al., 2018; Raj et al., 2020; Obermayer et al., 2022), and the resistance of employees to the adoption of Industry 4.0. Non-technological factors or human factors are the main issues ( Obermayer et al., 2022), because Industry 4.0, known for its complex interactions between machines and humans, requires compliance and solidarity among actors ( Robert et al., 2022). On the other hand, human involvement in the production process is decreasing as it is being replaced by machines ( Sony & Naik, 2020).

Barriers or challenges to the intention to implement Industry 4.0 also occur widely across various parts of the world (America, Europe, Asia) and in many sectors (organizations, online delivery, automotive, plastics industry, manufacturing, electricity, and others). These barriers are related to issues of attitude ( Cordero et al., 2023; Toni et al., 2021), social influence ( Ngan & Khoi, 2022), familiarity ( Tick et al., 2022), the TBL approach ( Nara et al., 2021), optimism ( Nabilah et al., 2020), consumer conformity ( Princes, 2020), the moderation of technology usage levels ( Saeedi et al., 2020), employee compliance and solidarity ( Robert et al., 2022), market uncertainty ( Prause, 2019), and communication problems ( Setiawan & Poerbosisworo, 2021).

Therefore, in the context of Industry 4.0 adoption, two important concepts that need to be considered are digital trust and sustainable attitudes. Digital trust refers to the belief that technology and information systems can be relied upon, secure, and well-integrated into business processes ( Launer et al., 2022). On the other hand, sustainable attitude involves awareness of the environmental and social impacts of business activities ( Chang et al., 2018), as well as a commitment to adopting environmentally responsible practices.

It is important to understand how uncertainty influences the relationship between key factors such as digital trust, sustainable attitude, and the intention to adopt industry 4.0. Additionally, previous research, conducted by Saeedi et al. (2020) and Toni et al. (2021), along with studies by Cordero et al. (2023) and Castillo-Vergara et al. (2022), has demonstrated the importance of measuring the level of Industry 4.0 adoption.

This study also uses perceived value as a mediating variable. Perceived value can be described as the significant exchange of benefits received (e.g., volume, quality, and convenience) and the sacrifices made to obtain those benefits (e.g., money, time, and effort). In the workplace, employees expect higher utility value from the adoption of new technology for their job performance, and they strive to achieve maximum results by considering limited situations and resources ( Walczuch et al., 2007).

Furthermore, supporting the transition towards Industry 4.0 is crucial, especially considering concerns about job loss and apprehensions regarding uncertainty, which may hinder the ability to adapt to the pace of new work rhythms or uncertainty avoidance. Thus, there is still a need for a deeper understanding of the mechanisms and implications in the context of adopting Industry 4.0. Therefore, this research aims to explore the influence of digital trust and sustainable attitude on perceived value and continuous intention to adopt Industry 4.0, moderated by uncertainty avoidance.

## 2. Literature Review and Hypothesis Development

### 2.1 Theory of Reasoned Action

This research follows the Theory of Reasoned Action (TRA) to construct the theoretical framework. In general, an increase in attitude and subjective norms leads to a stronger intention to perform a behavior ( Fishbein, 2008). Specifically, a favorable attitude towards the behavior and perceived social pressure to engage in it lead to stronger behavioral intentions. This theoretical framework is particularly relevant in understanding the adoption of Industry 4.0 technologies, where attitudes towards digital innovation and subjective norms regarding technological advancement play crucial roles in shaping individuals’ intentions to adopt these advancements.

### 2.2 Digital Trust

Kożuch (2021) elucidates that digital trust, a cornerstone of effective communication in digital organizations, embodies trust in the digital era. Unlike traditional trust, digital trust is distinguished by its reliance on technology. Paliszkiewicz & Chen (2022) further assert that cultivating digital trust stands as a paramount duty of management. It represents an intangible asset and a leadership skill vital for fostering a culture of mutual trust in the workplace and ensuring the proper management of IT assets and data. This conducive environment, termed as a facilitating condition, serves as the bedrock for the successful implementation of digital trust.

Digital trust serves as a pivotal catalyst for seamless digital interactions, gauging the expectations of an entity and validating its authenticity and reliability in digital transactions. Consequently, it has emerged as a prominent area of inquiry, particularly in the context of Industry 4.0 implementation. Studies delve into the impact of digital trust on the acceptance and utilization of Industry 4.0 technology by both organizations and individuals. Digital trust amalgamates traditional trust, underscored by human interactions, with digital technology, profoundly influencing a company’s credibility and growth trajectory.

The agility inherent in digital organizations underscores the critical role of human trust in conjunction with Industry 4.0, fostering organizational innovation performance—a facet closely aligned with the notion of digital trust ( Mubarak & Petraite, 2020), pertinent to this study. Additionally, primary sources such as ( Launer et al., 2022; Marcial & Launer, 2019; Saparudin et al., 2020; Abir et al., 2020; Arfi et al., 2021; Kim et al., 2021) offer invaluable insights into the multifaceted nature of digital trust.

### 2.3 Sustainable Attitude

The theory of attitudes discussed here focuses on attitudes towards sustainability, which encompasses environmental and economic aspects. Corporate sustainability, as described by Malek & Desai (2020), emphasizes meeting human resource needs while considering economic, social, and environmental factors. Elkington & Rowlands (1999) propose measuring corporate sustainability based on the Triple Bottom Line (TBL), comprising economic, social, and environmental factors. Pro-environmental behavior, studied through the Theory of Planned Behavior ( Ajzen, 1991), is influenced by sustainable attitudes and various situational factors ( Trail & McCullough, 2021). Krystallis et al. (2012) highlight cultural influences on sustainable attitudes towards food production. Chen (2015) suggests that sustainable attitudes influence intentions for pro-environmental practices, while Braun & McEachern (2010) emphasize their role in promoting such behavior. Gericke et al. (2019) identify economic, environmental, social, and international cooperation factors shaping sustainable awareness. Choi & Sirakaya (2005) develop the sustainable tourism attitude Scale to assess residents’ attitudes towards sustainable tourism, integrating various sustainability criteria. Michalos et al. (2012) stress the need for intensive education on sustainable development to improve knowledge and change attitudes and behaviors. Choi & Sirakaya (2005) measure sustainable attitudes using indicators such as environmental sustainability, social costs, and economic benefits, providing insights into promoting sustainable attitudes and behaviors.

### 2.4 Uncertainty Avoidance

After discussing digital trust and sustainable attitude, another variable that moderates the intention to adopt Industry 4.0 is the cultural dimension introduced by Hofstede (1980), namely uncertainty avoidance (UA). UA is one of the most frequently used cultural dimensions in technology adoption research, especially in online purchase intentions, adoption of big data or cloud storage, digital currency, local government e-government ( Elia, 2022), and mobile applications ( Negara et al., 2020). UA reflects individuals’ comfort with consistency, predictability, and strict rules versus tolerance for ambiguity and openness to change. This dimension has been widely studied in technology adoption research, particularly in online purchases, big data or cloud storage adoption, digital currency, e-government initiatives, green product preferences, and mobile applications. Individuals with low UA levels are more likely to accept and use new technology ( Negara et al., 2020). Despite its relevance, the moderation of UA towards Industry 4.0 adoption intentions remains underexplored, presenting a research gap in understanding its impact on technology adoption.

### 2.5 Perceived Value

According to McDougall & Levesque (2000), perceived value is an important indicator that can enhance consumer satisfaction and behavioral intentions. The concept of perceived value also determines consumers’ purchase intentions and decisions ( Zeithaml, 1988). Consumers typically weigh the benefits and drawbacks of a particular commodity within the constraints of their consumption capacity, purchase costs, and knowledge reserves, and then purchase the commodity with the greatest perceived value ( Nguyen et al., 2023). Perceived value is the standard used by consumers to measure the value of a product, and it is a subjective and personal evaluation made by users of the quality of a product or service on an emotional level ( Hapsari et al., 2016). The purpose of using the perceived value variable in this study is to test its role as a mediating variable between the independent variables digital trust and sustainable attitudes on behavioral information systems in the implementation of Industry 4.0, as a forefront of digital transformation programs ( Baron & Kenny, 1986).

### 2.6 Intention to Adopt Industry 4.0

The intention to adopt Industry 4.0 refers to an organization’s willingness and readiness to adopt and implement technologies and practices associated with Industry 4.0, such as cloud technology, Internet of Things (IoT), big data, simulation, autonomous robots, additive manufacturing, augmented reality, and business intelligence. This is a crucial factor in the adoption process as it reflects the organization’s commitment to technological advancement and changes in employee attitudes and leadership at strategic, tactical, and operational levels ( Cordero et al., 2023).

Various studies have employed theoretical frameworks such as the Theory of Planned Behavior (TPB) and Ajzen’s Theory of Planned Behavior to analyze the factors influencing the intention to adopt Industry 4.0 technology. Zaman et al. (2021) discovered that attitude, subjective norms, and perceived behavioral control positively impact the intention to adopt Industry 4.0 technology, particularly big data. Similarly, Perri et al. (2020) found that attitude, subjective norms, and perceived behavioral control positively affect the intention to adopt smart consumption and production behaviors. These studies offer valuable insights into the cognitive and affective beliefs that influence the intention to adopt Industry 4.0 technology.

### 2.7 Digital Trust, Sustainable Attitude, and Perceived Value

Digital trust, which is the users’ confidence in the safety, privacy, security, reliability, and ethical handling of data by companies in the digital environment, correlates with the perceived value of the information conveyed. The concept of trust has been proven to be a crucial factor influencing individuals’ perceptions of products, services, and technology. Various previous studies have indicated that trust in other contexts such as trust in online platforms (OPL) or trust in technology has a positive impact on perceived value ( Jayashankar et al., 2018; Konuk, 2018). In this regard, digital trust is viewed as a factor that can enhance the perceived value of the information provided by companies in the digital environment. Therefore, the hypothesis depicts the assumption that the higher the level of user trust in companies regarding digital aspects, the higher the value they attribute to the information received, as reflected in perceived value ( Mayer et al., 1995; Zhao & Chen, 2021).

In addition to digital trust, the hypothesis proposed is that there is a positive influence between sustainable attitudes and perceived value. Salehzadeh & Pool’s (2017) study on the relationship between brand attitudes and perceived value in the context of purchasing luxury goods showed that brand attitudes have a direct and significant impact on perceived value, as well as other dimensions of perception such as social, personal, and functional perceptions. Similarly, Charton-Vachet et al.’s (2020) study in France on the relationship between attitudes and intentions mediated by perceived value showed that customers’ attitudes toward purchasing intentions have a significant impact on perceived value. Both studies highlight the importance of perceived value in mediating the relationship between attitudes and purchase intentions. Therefore, based on a similar research framework, this hypothesis explores the possibility of a direct influence between sustainable attitudes and perceived value

H1.
*Digital trust positively and significantly affects perceived value.*

H2.
*Sustainable attitude positively and significantly affects perceived value.*

### 2.8 Digital Trust, Sustainable Attitude, and Intention to Adopt Industry 4.0

Saparudin et al. (2020) identified trust as the most influential factor affecting consumers’ intention to continue using mobile banking. Abir et al. (2020) discovered that eWOM, brand image, and trust significantly positively impact the intention to make online purchases. Contrarily, Arfi et al. (2021) found that performance expectation did not influence the intention to use IoT for eHealth. These studies offer insights for enhancing the design of connected devices, improving patient communication, and targeting potential users more effectively. Despite these findings, there is a lack of direct research on the relationship between Digital Trust and the Intention to Adopt Industry 4.0.

Cordero et al. (2023) surveyed organizations across several Latin American countries to identify drivers and barriers to Industry 4.0 adoption. Their findings underscored the pivotal role of quality technology in enhancing competitiveness and fostering national progress. Similarly, Castillo-Vergara et al. (2022) explored the technological acceptance of Industry 4.0 among rural Chilean students, revealing that factors such as technological optimism and facilitating conditions shape attitudes and intentions towards technology adoption. In contrast, Toni et al. (2021) utilized the TPB framework to analyze factors influencing the use of innovative vehicles, highlighting the importance of perceived behavioral control and subjective norms in determining intention, while attitude had no direct effect.

Saeedi et al. (2020) investigated the adoption of Industry 4.0 technology among dairy companies in Malaysia, revealing that the TPB accounted for a significant portion of the variation in intention to use such technology. Notably, they found that the moderating effect of technology usage on the influence of attitude on intention varied depending on usage levels. Similarly, Wang et al. (2018) demonstrated in China that environmental concern indirectly impacts the intention to adopt hybrid electric vehicles through various factors, including attitude, subjective norms, and perceived behavioral control.

H3.
*Digital trust positively and significantly affects intention to adopt.*

H4.
*Sustainable attitude positively and significantly affects intention to adopt.*

### 2.9 Perceived Value and Intention to Adopt Industry 4.0

Based on information from several previous studies, it is known that perceived value significantly influences the intention to adopt specific technologies or products. For example, in Miao et al.’s (2022) study on the relationship between perceived value and purchase intention through e-commerce, it was found that perceived value has a positive influence, albeit with the moderation of online purchase experience. This finding indicates a research gap regarding the relationship between perceived value and intention to adopt, especially with moderation. Similar findings occurred in Zhao & Chen’s (2021) study in China on the intention to purchase environmentally friendly homes, where higher perceived value led to a higher positive influence on purchase intention, with trust as a perceived value antecedent factor. Meanwhile, Yin & Qiu’s (2021) study on perceived value and purchase intention based on experiences with artificial intelligence showed that perceived value influences purchase intention with increasing technology accuracy. On the other hand, Hsu & Lin’s (2016) study on the Internet of Things (IoT) found that perceived value significantly influences users’ intention to use IoT services. Therefore, there is a conclusion that antecedent factors and moderation need to be considered to understand more deeply the influence of perceived value on intention to adopt.

H5.
*Perceived value positively and significantly affects intention to adopt.*

### 2.10 Uncertainty Avoidance as Moderator Variables

Based on the research provided, it can be hypothesized that uncertainty avoidance (UA) moderates the influence of digital trust and sustainable attitude on the intention to continue a relationship. Faqih (2022) found that uncertainty avoidance moderates the relationship between trust and behavioral intention in online shopping, particularly in cultures with high UA levels. Similarly, Ganguly & Nag (2021) highlighted the predictive role of UA in shaping trust and purchase intention in online shopping across different cultural values. Mosunmola et al. (2019) extended this by demonstrating that cultural dimensions, including UA, moderate the relationship between perceived value dimensions, trust, perceived risk, and purchase intention in online shopping. These insights emphasize the significant influence of UA in shaping trust and behavioral intention in online contexts. Therefore, drawing from these findings, it can be hypothesized that UA will moderate the relationship between digital trust and sustainable attitude and the intention to continue a relationship. However, direct research on this specific relationship in the context of Industry 4.0 adoption with the moderation of UA remains limited. Therefore, exploring this relationship would contribute to a deeper understanding of the interplay between cultural dimensions, digital trust, sustainable attitude, and intention to continue a relationship.

H6a.
*Uncertainty avoidance positively moderates the relationship among digital trust and intention to adopt.*

H6b.
*Uncertainty avoidance positively moderates the relationship among digital trust and intention to adopt.*

**Figure 1.**
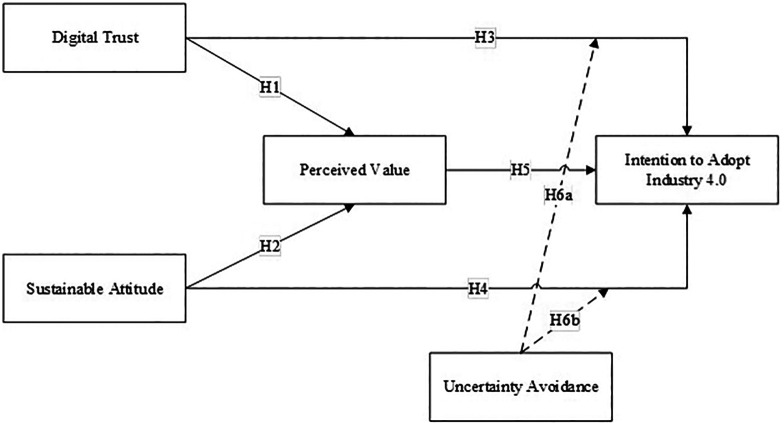
Conceptual Model. Source: author’s construct.

Based on the empirical study and literature review, the research conceptual model can be formulated as follows:

The conceptual framework of this research is illustrated in Figure 1. This model hypothesizes to explain H1: the relationship between Digital Trust and Perceived Value, H2: the relationship between Sustainable Attitude and Perceived Value, H3: the relationship between Digital Trust and Intention to Adopt Industry 4.0, H4: the relationship between Sustainable Attitude on Intention to Adopt Industry 4.0, H5: relationship between Perceived Value and Intention to Adopt Industry 4.0, H6a: The moderating role of Uncertainty Avoidance on the relationship between Digital Trust and Intention to Adopt Industry 4.0. H6b: The moderating role of Uncertainty Avoidance on the relationship between Sustainable Attitude and Intention to Adopt Industry 4.0.

## 3. Methods

The study adopts a quantitative approach, utilizing statistical analysis to analyze numerical research data. Data collection was conducted through a survey method, employing a questionnaire distributed online via Google Forms to gather primary data from respondents. In analyzing the data, Structural Equation Modeling (SEM) was employed using SmartPLS 3.2.4 software ( Ringle et al., 2014). This approach allows for a comprehensive examination of the relationships between variables, providing insights into the complex interplay of digital trust, sustainable attitudes, uncertainty avoidance, perceived value and intention to adopt industry 4.0 within the organizational context ( Ubaidillah et al., 2022).

The research was conducted at PT Schneider Indonesia, headquartered at Cibis Nine Building, Jalan TB Simatupang No.2, South Jakarta. This location was chosen because, despite having factories in Batam and Cikarang, only the employees at the headquarters are allowed to interact directly with customers. The research took approximately two months, with one month dedicated to data collection and another month for data processing. The study was carried out from 9 January to 9 February 2024, using primary data gathered from the assessments or perceptions of respondents. Surveys were conducted by distributing questionnaires online using Google Forms. In order to reduce the possibility of bias, attention was taken to ensure that the questionnaire was honestly created and thoroughly tested before being distributed. Anonymity was guaranteed to respondents in order to promote truthful responses.

The research sample comprises individuals representing variables such as digital trust, sustainable attitudes, uncertainty avoidance, perceived value and intention to adopt industry 4.0. The sample was drawn from employees of a prominent manufacturing company in Indonesia, recognized as a Light House Industry 4.0 by the World Economic Forum in Japan since 2019. This Paris-based company has consistently ranked among the most sustainable companies globally, as per Corporate Knight’s rankings over the past decade.

The selection of a manufacturing company as the research sample aligns with the focus on Industry 4.0 technology implementation within this sector. The population included all commercial office employees in Indonesia meeting the specified criteria. Employing the Systematic Random Sampling technique and based on the Slovin formula with a precision of e=5%, a sample of 189 employees was obtained, all of whom provided complete responses with no missing data.

Digital trust measurement draws from the works of Mubarak & Petraite (2020), including trust, internet of things, and smart factory, emphasizing the importance of trust in open innovation management within Industry 4.0. Similarly, sustainable attitudes measurement references Gericke et al. (2019), focusing on attitudes as integral components of sustainability consciousness, namely social, economy, and environment. Perceived value is measured through four indicators, namely emotional value, social value, professionalism value, and quality value ( Roig et al., 2006). Intention to adopt measurement references Khoa (2023) including continue using, alternative way, and stopping, while uncertainty avoidance is measured using Srite & Karahanna (2006) as the reference framework, namely regulations, order, job explanation, uncertain situation, and opportunity to innovate.

## 4. Results

Survey was conducted on 189 employees, all of whom met the specified criteria. All 189 data were collected and analyzed using IBM SPSS version 24 and SmartPLS 3.2.4, utilizing partial least squares structural equation modeling (PLS-SEM). Table 1 presents the results of the descriptive analysis of the research respondents.

**Table 1.** Respondent Characteristics.

Respondent Characteristics		Frequency	Percentage (%)
Age	24–33 years	37	19.58
	34–43 years	66	34.92
	44–53 years	71	37.57
	54–63 years	15	7.94
Gender	Male	132	69.84
	Female	57	30.16
Working period	1–6 years	15	7.94
	7–12 years	36	19.05
	13–18 years	73	38.62
	19–24 years	47	24.87
	25–30 years	18	9.52
Position	Supervisors	23	12.17
	Manager	62	32.80
	People Manager	22	11.64
	Staff	82	43.39
Education	High school	11	5.82
	Bachelors degree	164	86.77
	Masters degree	14	7.41
Program users	BFO	92	48.68
	SAP	60	31.75
	Others	37	19.58

Source: Field data (2024).

The characteristics of respondents are crucial to uncover because they depict employee profiles to understand their intention in adopting Industry 4.0. Analyzing respondent characteristics can serve as a basis for better strategic decision-making, enabling industries to allocate resources and efforts more effectively in implementing Industry 4.0 technology.

To investigate the influence of digital trust and sustainable attitude on the perceived value and continuous intention to adopt Industry 4.0, moderated by uncertainty avoidance, we employed the technique of Partial Least Squares Structural Equation Modeling (PLS-SEM). The PLS-SEM method was implemented using the SmartPLS 3.2.4 software.

### 4.1 Measurement Model Analysis

The measurement model comprises digital trust, sustainable attitude, uncertainty avoidance, perceived value, and intention to adopt, all structured reflectively. Assessing the model’s quality entails focusing solely on indicators with favorable and substantial factor loadings, while employing cronbach alpha, composite reliability, and average variance extracted (AVE) to evaluate the measurement model ( Patalay et al., 2015; Hair et al., 2011; Iqbal et al., 2021).

To measure reliability, we used cronbach’s alpha (CA) and composite reliability (CR). The results for CA and CR are presented in Table 2 for digital trust (0.904, 0.940), sustainable attitude (0.809, 0.888), uncertainty avoidance (0.809, 0.888), perceived value (0.892, 0.925), and intention to adopt (0.791, 0.918), where these values exceed 0.70, thus deemed acceptable and can proceed for further analysis ( Hair et al., 2011). Additionally, we tested convergent validity using the AVE values, namely digital trust (0.840), sustainable attitude (0.727), uncertainty avoidance (0.619), perceived value (0.755), and intention to adopt (0.630), all of which exceed the threshold of 0.50 ( Henseler et al., 2016). Furthermore, we assessed Fornell-Larcker for testing discriminant validity, as shown in Table 3.

**Table 2.** Measurement Model.

Variable	Code	Indicators	Loading	p-value	Cronbach Alpha	Composite Reliability	AVE
Digital Trust	DT1 DT2 DT3	Trust Internet of things Smart factory	0.973 0.872 0.901	<0.001 <0.001 <0.001	0.904	0.940	0.840
Sustainable Attitude	SA1 SA2 SA3	Social Economy Environment	0.768 0.909 0.875	<0.001 <0.001 <0.001	0.809	0.888	0.727
Uncertainty Avoidance	UA1 UA2 UA3 UA4 UA5 UA6	Regulations Order Job explanation Uncertain situation Opportunity to innovate Making changes	0.849 0.884 0.757 0.729 0.640 0.751	<0.001 <0.001 <0.001 <0.001 <0.001 <0.001	0.769	0.785	0.619
Perceived Value	PV1 PV2 PV3 PV4	Emotional value Social value Professionalism value Quality value	0.856 0.890 0.861 0.869	<0.001 <0.001 <0.001 <0.001	0.892	0.925	0.755
Intention to Adopt	IA1 IA2 IA3	Continue using Alternative way Stopping	0.947 0.933 0.850	<0.001 <0.001 <0.001	0.791	0.918	0.630

Source: Field data (2024).

**Table 3.** Discriminant Validity (Fornell-Larcker criteria).

	DT	SA	UA	PV	IA
DT	0.916				
SA	0.392	0.853			
UA	0.583	0.612	0.690		
PV	0.672	0.455	0.676	0.869	
IA	0.521	0.448	0.647	0.615	0.794

Source: Field data (2024).

Table 4 reveals that each variable demonstrates a stronger correlation with its respective construct compared to other constructs within the model. Consequently, it can be concluded that all variables exhibit discriminant validity, validating their suitability for subsequent analyses.

**Table 4.** Hypothesis Testing of SmartPLS Analysis.

Hypothesis	Relationship	Coefficients	p-value	Results
H1	DT ➔ PV	0.583	<0.001 **	Supported
H2	SA ➔ PV	0.226	<0.001 **	Supported
H3	DT ➔ IA	0.081	0.244 ^ns^	Not Supported
H4	SA ➔ IA	0.019	0.855 ^ns^	Not Supported
H5	PV ➔ IA	0.224	0.008 **	Supported
Moderating effects				
H6a	DT *UA ➔ IA	0.141	0.029 *	Supported
H6b	SA *UA ➔ IA	0.002	0.976 ^ns^	Not Supported

* Significant at 5%.

** Significant at 1%.

^ns^ Not significant.

### 4.2 Hypothesis Testing Results

**Figure 2.**
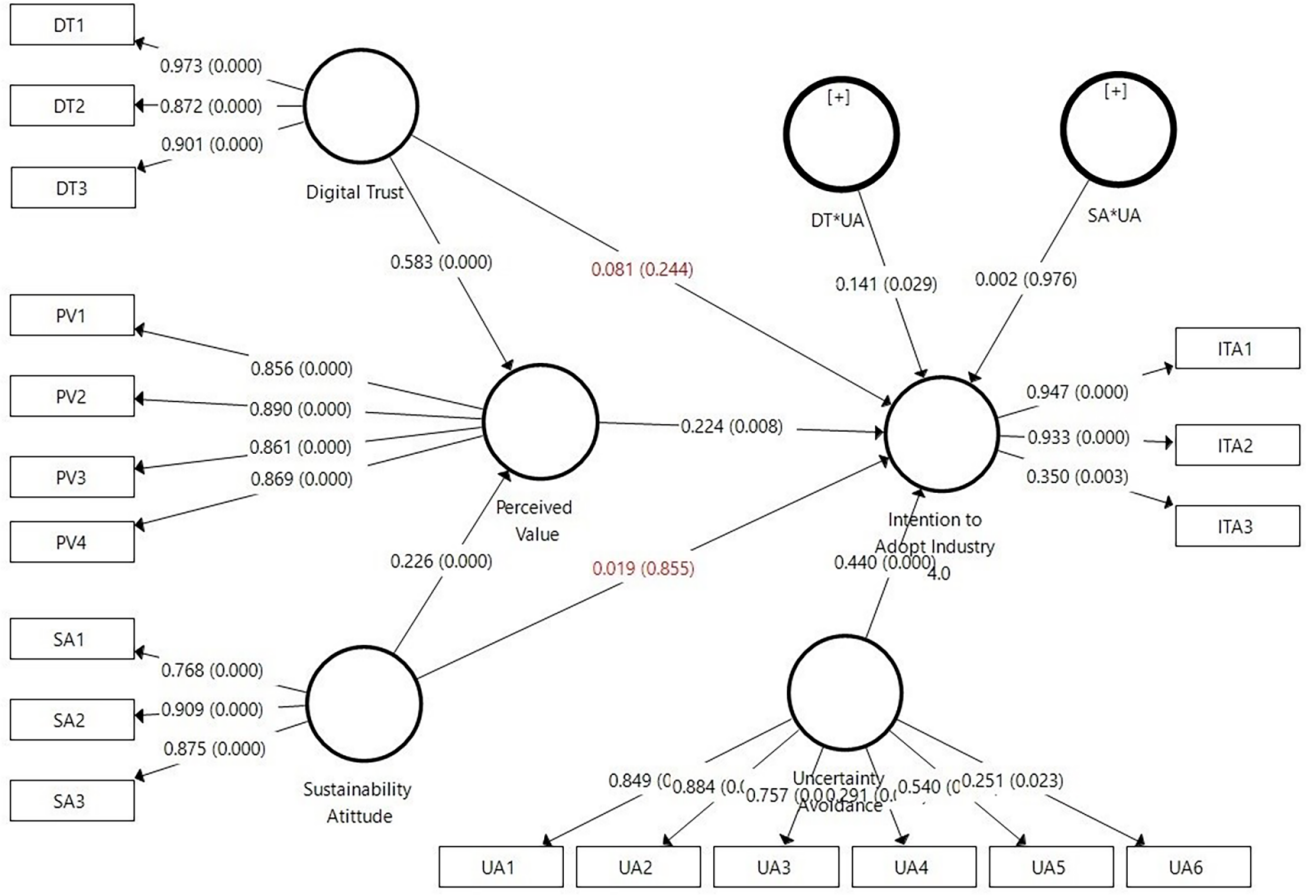
Structural Model Results Using SmartPLS.

The hypothesis testing results using PLS-SEM with SmartPLS indicate that H1 is accepted, where digital trust has a positive and significant effect on the perceived value (β=0.583, p≤0.001). H2 of this study is also accepted, where sustainable attitude has a positive and significant effect on the perceived value (β=0.226, p<0.001). However, H3 (β=0.081, p=0.224) and H4 (β=0.019, p=0.855) of this study are rejected because their p-values are greater than 0.05, indicating that digital trust and sustainable attitude do not significantly influence the intention to adopt Industry 4.0. Furthermore, H5 of the study shows the expected results, where perceived value has a positive and significant effect on the intention to adopt Industry 4.0 (β=0.224, p=0.008). Regarding the moderating effects, H6a stating that uncertainty avoidance positively moderates the relationship between digital trust and intention to adopt Industry 4.0 (β=0.141, p=0.029), is accepted. However, H6b of this study is rejected because uncertainty avoidance positively and insignificantly moderates the relationship between sustainable attitude and intention to adopt Industry 4.0 (β = 0.002, p = 0.976). Clearly, the results of hypothesis testing can be seen in Table 4 and Figure 2.

## 5. Discussion

### 5.1 The Relationship Between Digital Trust, Sustainable Attitude, and Perceived Value

The hypothesis testing results indicate that digital trust has a positive and significant influence on perceived value. Digital trust in this context refers to individuals’ level of trust in digital technology and platforms used. This finding is consistent with several previous studies, such as those conducted by Zhao & Chen (2021), Jayashankar et al. (2018), Konuk (2018), and Ponte et al. (2015), which also found a positive relationship between digital trust and perceived value. Strengthening digital trust can be key to enhancing the perceived value by customers, which in turn can increase customer loyalty and overall consumer satisfaction. Measures such as ensuring data security, transparency in technology use, and providing positive user experiences can help build and maintain strong digital trust ( Shin, 2019).

Additionally, this study indicates that sustainable attitude also has a positive and significant influence on perceived value. This finding is consistent with previous research by Charton-Vachet et al. (2020) and Salehzadeh & Pool (2017), which similarly identified a relationship between attitudes and perceived value. This indicates that a positive attitude towards sustainability issues can influence individuals’ perceived value of products or services. Consumers who have pro-sustainability attitudes tend to perceive the products or services as valuable because they consider the social and environmental impacts of their consumption ( Luchs & Kumar, 2017; Paswan et al., 2017). These findings have important implications in product marketing and business strategy development. Including sustainability aspects in marketing strategies and communicating the company’s commitment to sustainability can help enhance the perceived value of products or services in the eyes of consumers.

### 5.2 The Relationship Between Digital Trust, Sustainable Attitude, and Intention to Adopt Industry 4.0

This study reveals surprising findings that digital trust and sustainable attitude do not have a significant influence on intention to adopt. Despite previous research indicating the significant influence of digital trust ( Arfi et al., 2021; Kim et al., 2021; Saparudin et al., 2020; Rahman et al., 2020) and sustainable attitude ( Cordero et al., 2023; Castillo-Vergara et al., 2022; Wang et al., 2018; Toni et al., 2021; Saeedi et al., 2020) on intention to adopt in other contexts, these results indicate an interesting difference. The insignificance of the relationship between digital trust and sustainable attitude with intention to adopt suggests the complexity of factors influencing technology adoption. Although attitudes toward these solutions (in terms of social, economic, and environmental benefits) may be positive, these attitudes alone may not be sufficient to drive adoption intentions without considering subjective norms and perceived behavioral control. In addition, this finding also confirms the full mediation of perceived value. In the implementation of technology adoption, even though the company has decided on its Industry 4.0 sustainability strategy, it means that despite a positive attitude decision, emotional, social, professionalism, and quality value filters are still needed for this attitude decision to become a real action. The relationship between attitude and intention for sustainability is in line with Salas-Zapata et al.’s (2018) research.

These results have implications for both researchers and practitioners in the field of technology adoption and sustainability. They underscore the importance of considering a comprehensive set of factors when studying adoption behaviors and designing interventions to promote the uptake of new technologies. Future research may benefit from exploring the interplay between different factors and contextual variables to better understand the dynamics of technology adoption processes.

### 5.3 The Relationship Between Perceived Value and Intention to Adopt Industry 4.0

This study found that perceived value has a significant influence on intention to adopt, which aligns with the findings of several previous studies, such as Hsu & Lin (2016), Yin & Qiu (2021), Zhao & Chen (2021), and Miao et al. (2022). These results underscore the importance of individuals’ perceived value of a technology in determining their intention to adopt it. Most previous studies have emphasized the importance of perceived value in the context of technology adoption, particularly in terms of how the technology can provide benefits and added value to individuals or organizations. Therefore, these findings provide additional support to the previous conclusion that perceived value is a key factor influencing decisions to adopt new technology.

Additionally, this study also indicates that the relationship between perceived value and intention to adopt can be explained based on the concept of the TRA. This suggests that individuals’ perceptions of the value of a technology influence their intention to adopt it, consistent with the theory that emphasizes the significant role of attitudes and subjective norms in shaping behavior.

### 5.4 The Moderating Role of Uncertainty Avoidance

Although in direct influence, digital trust does not significantly affect intention to adopt Industry 4.0, uncertainty avoidance may serve as a moderating variable on this influence. The concept of uncertainty avoidance refers to the level of comfort individuals or societies have in dealing with uncertain or ambiguous situations. In the context of technology adoption, uncertainty avoidance can influence how individuals interpret and respond to levels of trust in digital technology.

The findings of this study support previous research conducted by Faqih (2022), Mosunmola et al. (2019), and Ganguly & Nag (2021), which suggest that uncertainty avoidance can act as a moderating variable between digital trust and intention to adopt. High levels of uncertainty avoidance tend to make individuals more cautious and seek certainty before they are willing to take risks in adopting new technology. These findings provide important contributions to our understanding of the complex dynamics influencing individuals’ intentions to adopt technology and can help design more effective strategies to encourage wider and faster technology adoption.

However, in the influence of sustainable attitude on intention to adopt, uncertainty avoidance did not significantly serve as a moderating variable. This study diverges from the findings of Sánchez-Franco et al. (2009), which illustrated a significant positive influence of attitudes on intention to adopt moderated by uncertainty avoidance. This difference can be attributed to cultural and psychological factors that influence how individuals respond to uncertainty in technology decision-making situations. When uncertainty avoidance is high, individuals tend to seek certainty and orderliness in decision-making. In terms of technology adoption, individuals’ attitudes toward technology become more critical, and they may be more inclined to consider other factors such as clearer benefits or more pressing needs.

Research by Lisana (2021) in Indonesia found that uncertainty avoidance is an important element of employees’ trust in using or not using technology. Therefore, in the application of research results related to the moderation of uncertainty avoidance, the proposal of rules ( Hong, 2020) regarding agreed-upon procedures and processes is needed, such as focusing on improving repetitive processes, adopting an account management approach, shared dashboard accuracy, dominant solution types, and dominant decision types.

## 6. Conclusion, Implication, and Limitation

This study highlights the importance of perceived value in influencing the intention to adopt Industry 4.0 technologies, as evidenced by significant findings consistent with prior research. Moreover, the study underscores that both digital trust and sustainable attitude significantly influence perceived value, contributing to the understanding of the adoption process. However, it also reveals surprising results regarding the insignificant direct influence of digital trust and sustainable attitude on intention to adopt. This finding also confirms the full mediation of perceived value, it means that despite a positive attitude decision, emotional, social, professionalism, and quality value filters are still needed for this attitude decision to become a real action of technology adoption. The moderating role of uncertainty avoidance yields expected results on the relationship between digital trust and intention to adopt. However, not on the relationship between sustainable attitude and intention to adopt, shedding light on the complex dynamics of technology adoption. It will need agreed-upon procedures and processes, such as focusing on improving repetitive processes, adopting an account management approach, shared dashboard accuracy, dominant solution types, and dominant decision types.

Understanding the significance of perceived value in driving adoption intentions underscores the importance of emphasizing the benefits and value propositions of Industry 4.0 technologies. Building digital trust and fostering sustainable attitudes remain important endeavors, although their direct influence on adoption intentions may be less pronounced. Practitioners should consider incorporating strategies to mitigate uncertainty avoidance, such as providing clear information and reducing perceived risk.

One limitation of this study is its reliance on self-reported data, which may introduce response bias. Additionally, the study focused on a specific context and population, limiting the generalizability of the findings. Future research could address these limitations by employing diverse methodologies and examining different cultural contexts. Additionally, future research could explore additional moderators of the relationship between digital trust, sustainable attitude, and intention to adopt, considering factors such as cultural differences and organizational contexts. Longitudinal studies could provide insights into the dynamics of technology adoption over time.

### Ethical statement

I have adhered to the writing guidelines and ethics of the F1000Research journal. Based on letter number 455/UN10.F03/PP/2024 regarding the Research Permit Application, my institution granted permission for the research on January 8, 2024. Additionally, I have received a research permit letter from PT Schneider Indonesia to conduct research at the company.

Participants’ consent for this study was obtained verbally, as approved by the Institutional Review Board at Brawijaya University. Verbal consent was chosen due to the practicalities of conducting the study remotely and ensuring timely participation. The ethics committee reviewed and approved this method to maintain participant confidentiality and streamline the data collection process.

## Author contributions

Conceptualization, Y.S., E.S.A., W.W. and T.N.; methodology, Y.S. and T.N.; software, Y.S.; validation, E.S.A., W.W. and T.N.; formal analysis, Y.S.; investigation, W.W.; resources, Y.S.; data curation, T.N.; writing—original draft preparation, Y.S.; writing—review and editing, Y.S.; visualization, Y.S.; supervision, E.S.A., W.W. and T.N.; project administration, Y.S.; funding acquisition, Y.S. All authors have read and agreed to the published version of the manuscript.

## Data Availability

The data presented in this study are available on request from the corresponding author due to confidentiality agreements with the participants involved in the research. Our data statement is complete and adheres to the journal’s guidelines. Access to the data is restricted to protect participant privacy. Researchers wishing to access the data must submit a formal request to the corresponding author, detailing the purpose of their research, the specific data needed, intended use, and measures for ensuring data security and participant confidentiality. Requests will be evaluated on a case-by-case basis, and access will be granted under specific conditions approved by our Institutional Review Board (IRB). For further inquiries, please contact yulisartono@student.ub.ac.id. Figshare: Checklist for “Sustainable Digital Transformation: Its Impact on Perceived Value and Adoption Intention of Industry 4.0 in Moderating Effects of Uncertainty Avoidance”, DOI: https://doi.org/10.6084/m9.figshare.26302273.v2 ( Sartono et al., 2024). Data are available under the terms of the Creative Commons Attribution 4.0 International license (CC-BY 4.0) STROBE checklist for “Sustainable Digital Transformation: Its Impact on Perceived Value and Adoption Intention of Industry 4.0 in Moderating Effects of Uncertainty Avoidance”, https://doi.org/10.6084/m9.figshare.26302273.v2 ( Sartono et al., 2024).
