# Supplementary material for: Sustainable Digital Transformation: Its Impact on Perceived Value and Adoption Intention of Industry 4.0 in Moderating Effects of Uncertainty Avoidance
Source: F1000Res. 2024 Sep 13;13:821. Originally published 2024 Jul 22. [Version 2] doi: 10.12688/f1000research.152228.2 (PMC11369593; doi:10.12688/f1000research.152228.2)
Supplement: Peer review history

# Reviewer report: Reviewer response for version 2

**DOI:** 10.5256/f1000research.171417.r323343

**Published:** 2024-09-16

**Authors:** Djoko Budiyanto Setyohadi

**Affiliations:** 1 Atmajaya University, Yogyakarta, Indonesia

I agree with authors revision.

Is the work clearly and accurately presented and does it cite the current literature?

Yes

If applicable, is the statistical analysis and its interpretation appropriate?

Yes

Are all the source data underlying the results available to ensure full reproducibility?

Yes

Is the study design appropriate and is the work technically sound?

No

Are the conclusions drawn adequately supported by the results?

Yes

Are sufficient details of methods and analysis provided to allow replication by others?

No

Reviewer Expertise:

Socio informatics, information system, and user interface user experience

I confirm that I have read this submission and believe that I have an appropriate level of expertise to confirm that it is of an acceptable scientific standard.

# Reviewer report: Reviewer response for version 2

**DOI:** 10.5256/f1000research.171417.r323342

**Published:** 2024-09-14

**Authors:** Muhammad Faraz Mubarak

**Affiliations:** 1 Dalhousie University, Halifax, Nova Scotia, Canada

.No further comments

Is the work clearly and accurately presented and does it cite the current literature?

Yes

If applicable, is the statistical analysis and its interpretation appropriate?

Yes

Are all the source data underlying the results available to ensure full reproducibility?

Partly

Is the study design appropriate and is the work technically sound?

Yes

Are the conclusions drawn adequately supported by the results?

Yes

Are sufficient details of methods and analysis provided to allow replication by others?

Yes

Reviewer Expertise:

innovation management > digital transformation-->collaborative innovation

I confirm that I have read this submission and believe that I have an appropriate level of expertise to confirm that it is of an acceptable scientific standard.

# Reviewer report: Reviewer response for version 1

**DOI:** 10.5256/f1000research.166963.r316104

**Published:** 2024-09-02

**Authors:** Djoko Budiyanto Setyohadi

**Affiliations:** 1 Atmajaya University, Yogyakarta, Indonesia

The paper entitled "Sustainable Digital Transformation: Its Impact on Perceived Value and Adoption Intention of Industry 4.0 in Moderating Effects of Uncertainty Avoidance" investigates the mechanisms and implications of adopting Industry 4.0. It examines how digital trust and a sustainable attitude influence perceived value and the ongoing intention to adopt Industry 4.0. The paper addresses an engaging topic and presents a well-executed experiment, supported by a solid theoretical foundation. This makes it a valuable resource for both practitioners and academics. However, there are points that require improvement before the paper can be accepted for indexing.

- The claim that “there is still a need for a deeper understanding of the mechanisms and implications in the context of adopting Industry 4.0” is intriguing but lacks clear elaboration. The paper should better clarify the relationships among the variables in the proposed model.
- The literature review is well-structured but could benefit from additional context, especially in relation to the area of the study. It would be beneficial to include more sources that address similar technological acceptance issues, particularly in the Indonesian context.
- The conclusion should be expanded to reflect the key claims made by the author.

In summary, the paper is interesting and useful for the reader related to adopting Industry 4.0. It requires further development and extension. It could be considered for indexing once revisions addressing the above points are made.

Is the work clearly and accurately presented and does it cite the current literature?

Yes

If applicable, is the statistical analysis and its interpretation appropriate?

Yes

Are all the source data underlying the results available to ensure full reproducibility?

Yes

Is the study design appropriate and is the work technically sound?

No

Are the conclusions drawn adequately supported by the results?

Yes

Are sufficient details of methods and analysis provided to allow replication by others?

No

Reviewer Expertise:

Socio informatics, information system, and user interface user experience

I confirm that I have read this submission and believe that I have an appropriate level of expertise to confirm that it is of an acceptable scientific standard.

# Response

**Published:** 2024-09-03

**Authors:** Yuli Sartono

**Affiliations:** Business Administration, Universitas Brawijaya, Malang, East Java, Indonesia

Thank you for your thoughtful and constructive feedback on our research paper titled "Sustainable Digital Transformation: Its Impact on Perceived Value and Adoption Intention of Industry 4.0 in Moderating Effects of Uncertainty Avoidance." We are pleased to hear that you find the topic engaging and the study valuable for both practitioners and academics. We appreciate your detailed comments, which have guided us in improving the quality of our paper. Below, we address each of your points:

1. Clarification of the Need for Deeper Understanding: We have now elaborated on the statement regarding the need for a deeper understanding of the mechanisms and implications in adopting Industry 4.0. In the revised paper, we have clarified how the existing literature gaps relate to our study’s objectives. We provide a more detailed explanation of the specific mechanisms, highlighting the importance of understanding these relationships to advance Industry 4.0 adoption.
2. Enhancing the Literature Review with Additional Context: In response to your suggestion, we have enriched the literature review by including more recent studies that address similar technological acceptance issues, particularly within the Indonesian context. These additions provide a broader understanding of how local cultural factors impact the adoption of Industry 4.0 technologies, ensuring that the review reflects the most current and relevant research in this area.
3. Expansion of the Conclusion: We have expanded the conclusion to better reflect the key claims made throughout the paper. The revised conclusion now offers a comprehensive summary of our findings and ties them back to the original research questions and hypotheses. This ensures that the significance of our findings is clearly articulated and underscores the contributions of our study to the field of Industry 4.0 and digital transformation.

We are committed to enhancing the clarity, depth, and rigor of our paper. Your feedback has been instrumental in guiding us to make the necessary revisions that improve the quality and impact of our study. We believe that by addressing these points, our paper now offers a more robust and valuable contribution to the academic community and industry practitioners.

 Thank you once again for your insightful comments and suggestions.

 Sincerely,

 Authors

# Reviewer report: Reviewer response for version 1

**DOI:** 10.5256/f1000research.166963.r306607

**Published:** 2024-08-29

**Authors:** Muhammad Faraz Mubarak

**Affiliations:** 1 Dalhousie University, Halifax, Nova Scotia, Canada

The study provides interesting insights where role of digital trust and sustainable attitude is tested towards perceived value, that is further assumed towards intention to adopt i4.0. The study is based on reasonable theoretical background, as well as hypothesis development is considerable. 

 The methodology applied is also suitable, and the context is acceptable tool. Further, results are based on robust analysis to support and reject respective hypothesis. Having said that, the overall study looks good, however, authors are suggested to highlight the originality and novelty of study, though it has solid foundations and USPs such as digital trust and sustainable attitude towards perceived value and then intention (with a mdoerator of course). I wish all the best to all co-authors and grateful for providing me an opportunity to quickly read this very interesting piece of research.

Is the work clearly and accurately presented and does it cite the current literature?

Yes

If applicable, is the statistical analysis and its interpretation appropriate?

Yes

Are all the source data underlying the results available to ensure full reproducibility?

Partly

Is the study design appropriate and is the work technically sound?

Yes

Are the conclusions drawn adequately supported by the results?

Yes

Are sufficient details of methods and analysis provided to allow replication by others?

Yes

Reviewer Expertise:

innovation management > digital transformation-->collaborative innovation

I confirm that I have read this submission and believe that I have an appropriate level of expertise to confirm that it is of an acceptable scientific standard.

# Response

**Published:** 2024-09-03

**Authors:** Yuli Sartono

**Affiliations:** Business Administration, Universitas Brawijaya, Malang, East Java, Indonesia

Dear Reviewer,

 Thank you for your thorough review of our manuscript titled "Sustainable Digital Transformation: Its Impact on Perceived Value and Adoption Intention of Industry 4.0 in Moderating Effects of Uncertainty Avoidance." We greatly appreciate your positive feedback on our study's theoretical background, hypothesis development, methodology, and robust analysis. We are pleased that you found our research interesting and valuable.

 We acknowledge your suggestion to further highlight the originality and novelty of our study, particularly concerning the unique aspects of digital trust and sustainable attitude in relation to perceived value and intention to adopt Industry 4.0 technologies. In response, we will enhance the discussion section to better emphasize these points and clarify how our research contributes to the existing body of knowledge in this field.

 Regarding the availability of source data, we understand the importance of data accessibility for reproducibility. As mentioned, our data is subject to confidentiality agreements, but we will outline a clear process for requesting access and consider anonymizing the data wherever possible. This should facilitate replication while protecting participant confidentiality.

 Once again, we appreciate your insightful comments and suggestions, which have guided us in improving the quality of our manuscript. We are committed to addressing your feedback and ensuring that our study provides a meaningful contribution to the field of digital transformation and Industry 4.0 adoption.

 Thank you for your time and expertise in reviewing our work. We look forward to your continued feedback and suggestions.

 Sincerely,

 Authors

# Reviewer report: Reviewer response for version 1

**DOI:** 10.5256/f1000research.166963.r306602

**Published:** 2024-07-25

**Authors:** Mohamad Zreik

**Affiliations:** 1 Sun Yat-sen University, Zhuhai, China

**Summary of the Article**

 The research article titled *"Sustainable Digital Transformation: Its Impact on Perceived Value and Adoption Intention of Industry 4.0 in Moderating Effects of Uncertainty Avoidance" *explores the relationships between digital trust, sustainable attitude, perceived value, and the intention to adopt Industry 4.0 technologies. The study also examines how uncertainty avoidance moderates these relationships. Data were collected from 189 employees at leading manufacturing companies in Indonesia recognized for their Industry 4.0 practices. The findings reveal that digital trust and sustainable attitude significantly influence perceived value, but do not directly affect the intention to adopt Industry 4.0 technologies. Uncertainty avoidance moderates the relationship between digital trust and adoption intention.

 Detailed Analysis

1. **Presentation and Citation of Current Literature**
  - **Partly**
    - The work is clearly presented and provides a comprehensive analysis. However, some references are outdated. Incorporating more recent studies would strengthen the paper's relevance and ensure it reflects the latest developments in Industry 4.0 and digital transformation.
    - **Recommendation**: Update the literature review to include more recent sources from the past five years to ensure the study is grounded in the latest research.
2. **Study Design Appropriateness and Technical Soundness**
  - **Yes**
    - The study design is appropriate for the research questions posed. The use of Partial Least Squares (PLS) methodology with SmartPLS software is suitable for testing the proposed hypotheses and exploring moderating effects. The sample selection and data collection methods are clearly described, and the statistical analysis is robust, providing valid and reliable results.
3. **Details of Methods and Analysis for Replication**
  - **Yes**
    - The study provides sufficient details of the methods and analysis to allow replication. It describes the data collection process, sample selection, survey methodology, and the use of SmartPLS software for PLS-SEM. The measurement of variables, hypothesis testing, and statistical analysis procedures are detailed adequately for replication.
4. **Statistical Analysis and Interpretation**
  - **Yes**
    - The statistical analysis and its interpretation are appropriate. The use of PLS-SEM is well-suited for the study's objectives, and the analysis includes reliability and validity assessments, hypothesis testing, and the examination of moderating effects. The interpretation of results is consistent with the data and supports the study's conclusions.
5. **Availability of Source Data**
  - **Partly**
    - While the study mentions that data are available on request from the corresponding author due to confidentiality agreements, this does not ensure full reproducibility. Ideally, the source data should be openly accessible or provided in a manner that allows independent verification and replication of the results without additional barriers.
    - **Recommendation**: Provide a more detailed data availability statement and consider anonymizing the data to make it fully accessible. If confidentiality agreements are a constraint, clearly outline the process for requesting data access.
6. **Conclusions Supported by Results**
  - **Yes**
    - The conclusions drawn are adequately supported by the results. The findings align with the hypotheses and statistical analysis, and the discussion appropriately interprets the results in the context of the research questions. The conclusions are based on the data presented and provide valuable insights into the impact of digital trust, sustainable attitude, and uncertainty avoidance on the adoption of Industry 4.0 technologies.

Constructive Feedback for Improvement

1. **Update Literature Review**
  - Incorporate more recent studies (from the past five years) to ensure the research is current and reflects the latest developments in Industry 4.0 and digital transformation.
2. **Enhance Data Accessibility**
  - Provide a more detailed data availability statement. Consider anonymizing the data to make it fully accessible. If confidentiality is an issue, outline a clear process for requesting data access, ensuring that the steps are not overly cumbersome for researchers wishing to replicate the study.
3. **Expand Discussion on Non-significant Findings**
  - Provide a deeper exploration of why digital trust and sustainable attitude did not directly affect the intention to adopt Industry 4.0 technologies. Discuss potential reasons for these findings and suggest areas for future research to explore these relationships further.
4. **Address Cultural Dimensions More Thoroughly**
  - Given the significant role of uncertainty avoidance, consider a more detailed discussion of cultural dimensions and their impact on technology adoption. This could include a comparison with findings from different cultural contexts to provide a broader understanding of the role of cultural factors.

By addressing these points, the authors can enhance the scientific soundness and impact of their study, making it more robust and valuable to the academic community and practitioners in the field of Industry 4.0 and digital transformation.

Is the work clearly and accurately presented and does it cite the current literature?

Partly

If applicable, is the statistical analysis and its interpretation appropriate?

Yes

Are all the source data underlying the results available to ensure full reproducibility?

Partly

Is the study design appropriate and is the work technically sound?

Yes

Are the conclusions drawn adequately supported by the results?

Yes

Are sufficient details of methods and analysis provided to allow replication by others?

Yes

Reviewer Expertise:

-

I confirm that I have read this submission and believe that I have an appropriate level of expertise to confirm that it is of an acceptable scientific standard, however I have significant reservations, as outlined above.

# Response

**Published:** 2024-09-03

**Authors:** Yuli Sartono

**Affiliations:** Business Administration, Universitas Brawijaya, Malang, East Java, Indonesia

Dear Reviewer,

 Thank you for your detailed and constructive feedback on our research article titled "Sustainable Digital Transformation: Its Impact on Perceived Value and Adoption Intention of Industry 4.0 in Moderating Effects of Uncertainty Avoidance." We appreciate the time and effort you have taken to review our work and provide insightful comments to enhance its quality. Below, we address each of your points:

1. **Updating the Literature Review:** We appreciate your suggestion to include more recent studies in the literature review. We have now updated this section to incorporate studies from the past five years, ensuring that our literature review reflects the latest developments in Industry 4.0 and digital transformation. This revision helps in grounding our work in the most current research and provides a comprehensive overview of relevant literature.
2. **Enhancing Data Accessibility:** We understand the importance of data accessibility for reproducibility and transparency. However, our data are subject to confidentiality agreements with the participants and restrictions imposed by the institution where the research was conducted. To address this concern, we will revise our data availability statement to outline a clear and streamlined process for requesting data access. While we may not be able to make the data fully accessible due to confidentiality issues, we will consider anonymizing the data wherever possible. We will ensure that the process for requesting data access is straightforward and not overly cumbersome, enabling other researchers to replicate our study while maintaining the confidentiality of our participants.
3. **Expanding the Discussion on Non-significant Findings:** We have revised the discussion section to provide a deeper exploration of the non-significant findings, specifically why digital trust and sustainable attitude did not directly affect the intention to adopt Industry 4.0 technologies. The revised discussion now includes potential explanations and contextual factors that might have influenced these results. We have also suggested future research directions to further investigate these relationships.
4. **Addressing Cultural Dimensions More Thoroughly:** In response to your feedback, we have expanded our discussion on cultural dimensions, particularly the role of uncertainty avoidance, in shaping technology adoption behaviors. We have included a more detailed analysis of how cultural factors influence the adoption of Industry 4.0 technologies. Additionally, we have compared our findings with studies conducted in different cultural contexts to provide a broader understanding of the impact of cultural dimensions on technology adoption.

We are committed to improving the scientific rigor and impact of our study, and we believe that incorporating your feedback will significantly enhance its value to the academic community and practitioners in the field of Industry 4.0 and digital transformation. We appreciate your constructive suggestions and will make the necessary revisions to address them.

 Thank you once again for your valuable feedback.

 Sincerely,

 Authors
